# Immediate effects of photobiomodulation on maximum pressure and endurance of the tongue in adults: a randomized clinical trial

**DOI:** 10.1590/2317-1782/e20240317en

**Published:** 2026-01-26

**Authors:** Lara Kristine Silva Gomes Campos Soares, Lorena Freitas Coelho, Renata Maria Moreira Moraes Furlan, Mariana Rodrigues Batista

**Affiliations:** 1 Graduação em Fonoaudiologia, Universidade Vale do Rio Doce – UNIVALE - Governador Valadares (MG), Brasil.; 2 Departamento de Fonoaudiologia, Universidade Federal de Minas Gerais – UFMG - Belo Horizonte (MG), Brasil.; 3 Departamento de Fonoaudiologia, Universidade Vale do Rio Doce – UNIVALE - Governador Valadares (MG), Brasil.

**Keywords:** Muscle Strength, Physical Endurance, Laser Therapy, Tongue, Stomatognathic System

## Abstract

**Purpose:**

To verify whether there are immediate effects of photobiomodulation on maximum tongue pressure and endurance.

**Methods:**

This was a double-blind experimental study that investigated the immediate effects of photobiomodulation on maximum tongue pressure and endurance in individuals without functional alterations of this structure. The non-probabilistic sample consisted of 60 individuals of both sexes, aged between 18 and 35 years, divided into four groups. The tested doses were 7, 9, and 11J per point, using infrared wavelength, applied to six points on the dorsal surface of the tongue (three longitudinal points in two rows) and three longitudinal points on each lateral side, totaling 12 application points. The placebo group underwent the same procedures as the others, but the device was not activated. Participants underwent an intraoral evaluation of the tongue using the MBGR protocol to determine eligibility, as well as maximum tongue pressure and endurance assessment using the IOPI, both before and after irradiation. The maximum tongue pressure and endurance were compared before and after photobiomodulation.

**Results:**

The groups were homogeneous regarding sex, age, maximum tongue pressure, and endurance before irradiation. No differences were observed in tongue pressure or resistance between the pre and post-irradiation moments in any of the tested groups.

**Conclusion:**

Photobiomodulation, at the tested doses, did not produce immediate effects on maximum tongue pressure or resistance in adults without structural and/or functional alterations of the tongue.

## INTRODUCTION

The stomatognathic system is made up of structures complexly related to the functions of sucking, chewing, swallowing, breathing, and speaking^(1)^. They are vital human functions; hence, any structural alteration may consequently disrupt this system^(2)^.

The tongue is an organ composed of intrinsic and extrinsic muscles arranged in such a way that its movements allow the performance of orofacial functions^(3,4)^.

Some clinical conditions, such as Parkinson's disease^(5)^, mouth breathing^(6)^, Down syndrome^(7)^, and amyotrophic lateral sclerosis^(8)^, compromise tongue muscle pressure and endurance. Speech-language pathologists (SLPs) work in the prevention, evaluation, diagnosis, and treatment of alterations that may affect stomatognathic functions. Speech-language-hearing pathologists are increasingly interested in using photobiomodulation (PBM) as a therapeutic resource^(9)^ because it is a noninvasive, painless technique with low risk to the patient, no side effects^(10)^, and no reports of toxicity^(9)^. PBM can promote benefits for muscle tissue, including improved muscle performance, increased strength gain, and muscle relaxation^(11)^.

PBM consists of the application of light to a biological system capable of stimulating the beginning of a photochemical process, seen more actively in mitochondria, increasing the production of adenosine triphosphate (ATP)^(12)^, in addition to other molecular mechanisms of cell proliferation. It increases interleukins, synthesis of deoxyribonucleic acid (DNA), reactive oxygen species, cytochrome c-oxidase, and so forth^(13)^, favoring cell metabolism, and potentially generating effects such as analgesia, tissue repair, and reduction of muscle fatigue, among others^(9)^.

Recent studies have investigated the immediate effects of PBM on the pressure^(14)^ and electromyographic fatigue of the lips^(10)^. The results showed an immediate increase in lip pressure after PBM at a wavelength of 808 nm, with 7 J at six points around the lips, for a total dose of 42 J^(14)^. No effects on electromyographic fatigue were observed in the orbicularis oris muscle, which used a dose of 4 J per point at wavelengths of 660 and 808 nm^(10)^.

Radiation can have photochemical effects within minutes, known as immediate effects^(15)^.

There are no well-defined dosimetry protocols for each case involving orofacial muscles^(11)^. It is the professional performing the photobiomodulation therapy who will define which light wavelength and dose will be used, as well as the irradiation points. Therefore, they must understand the consequent effects of each dose, wavelength, and application points. This makes the use of this therapeutic resource challenging, as there is no consensus on the ideal parameters, nor on protocols targeted to each objective^(11)^ in oral-motor therapy. Hence, further studies are needed to create targeted PBM protocols for orofacial muscles.

Few studies have addressed the effects of PBM on orofacial muscles^(11,12)^, and they focus specifically on the orbicularis oris muscle. It is known that photobiomodulation has photochemical effects. To date, no study has investigated the effects of photobiomodulation on tongue muscles.

Thus, the main objective of this study was to determine whether PBM has immediate effects on maximum tongue pressure and endurance.

## METHODS

This randomized, double-blind clinical trial investigated the immediate effects of PBM on maximum tongue pressure and endurance in subjects without functional or structural tongue alterations.

Data were collected at Vale do Rio Doce University. The project was approved by the institution's Research Ethics Committee (approval no. 6.854.040) and published in the Brazilian Clinical Trials Registry (ReBEC) under number RBR-10bf7yj6.

The non-probabilistic sample consisted of 60 individuals without functional or structural tongue alterations, of both sexes, with a mean age of 21.1 years, a minimum of 18, a maximum of 34 years, and a standard deviation of 2.9 years. Participants were randomly divided into four groups: group 1 (G1), group 2 (G2), group 3 (G3), and a placebo group (PG). Participants took a piece of paper with a number (from 1 to 60) from a box. The numbers belonging to each intervention group had been previously defined.

- G1: group subjected to PBM at a wavelength of 808 nm (infrared), with 7 J per point, totaling 84 J.
- G2: group subjected to PBM at a wavelength of 808 nm (infrared), with 9 J per point, totaling 108 J.
- G3: group subjected to PBM at a wavelength of 808 nm (infrared), with 11 J per point, totaling 132 J.
- PG: group subjected to the same procedure as the participants in G1, G2, and G3, without activating the equipment.

The sample size was defined based on previous studies that evaluated the immediate effects of PBM on orofacial muscles^(10-12)^, and no sample size calculation was performed. Students, faculty, and staff of the institution who met the inclusion criteria were invited to participate in the study. The sample included individuals of both sexes, aged 18 to 35 years, who agreed to participate in the research by signing an informed consent form, with no cognitive alterations (they had to be able to follow the commands and tasks; if they were unable to understand/comply with the commands, they would not be included), no functional and/or structural alterations of the tongue, no oral lesions that caused pain or discomfort, lingual frenulum with fixations in the middle third of the tongue and in the sublingual caruncle (verified through the intraoral examination, specifically of the tongue, of the Myofunctional Assessment Protocol [MBGR] protocol), no neurogenic diseases (that would affect their understanding of or compliance with commands), and who did not present contraindications for phototherapy. Contraindications were assessed through a questionnaire, according to the equipment manufacturer's manual and specific literature. These included photosensitivity, pregnancy, glaucoma, undiagnosed lesions on or near the irradiated area, infection at the injection site, history of cancer, and use of a pacemaker or other electronic implant. Other inclusion criteria were not taking medications that could cause muscle weakness^(16)^ in the 48 hours prior to data collection and reporting allergies to the materials used. These data were collected during the initial interview. Exclusion criteria were failure to perform all proposed tasks and intolerance to keeping the IOPI (Iowa Oral Performance Instrument) bulb in the oral cavity.

After signing an informed consent form, the participant was instructed to remain seated in a chair, guided by the Frankfurt Plane, maintaining an upright posture and 90° flexion between the ankle, knee, and hip. A trained researcher with experience in the treatment of orofacial myofunctional disorders performed the intraoral MBGR^(17)^ examination, specifically of the tongue, to identify and exclude individuals with alterations in this organ.

Maximum pressure and endurance were assessed using an instrument that presents numerical values ​​for each parameter evaluated. The instrument used was the IOPI , which consists of an air bulb (3.5 cm long and 1 cm in diameter), a pressure transducer, a 1.5 cm plastic tube connecting the bulb to the transducer, and an LCD screen. The IOPI bulb was positioned in two regions: first, in the anterior region of the tongue, just behind the alveolar papilla, and second, in the posterior region, with its anterior limit parallel to the beginning of the first molars^(18)^. After positioning the instrument in the anterior region, the participant was asked to press the tongue toward the palate with the greatest possible force for 2 seconds. This procedure was performed three times, with a 1-minute interval, and the maximum pressure was the arithmetic mean of all maximum pressure peaks. Then, the bulb was positioned in the posterior region of the tongue, and the participant was instructed to repeat the movement for the same amount of time.

The bulb was positioned in the same way to assess tongue endurance as for maximum tongue pressure. However, the participant was instructed to maintain the pressure for as long as possible. To ensure the participant's pressure, they relied on the IOPI's own biofeedback, which illuminates green when the individual reaches the pre-programmed pressure (a value entered by the evaluator into the IOPI, which is half the maximum pressure value). One collection was made from the anterior region and another from the posterior region of the tongue, with a 10-minute interval between measurements.

After the initial assessment, participants were randomly assigned to intervention groups. PBM was performed using a 100 mW MMOptics^®^ Laser Duo device. The irradiation parameters are described in Chart 1.

**Chart 1:** Laser parameters

**Irradiation parameters**	**Values**
Wavelength	808 nm (infrared)
Operating mode	Continuous
Optical output	100 mW
Beam output diameter	1.95 mm
Beam output area	0.03 cm^2^
Power density	3.3 W/cm^2^
Energy per point	7 J
Energy density per point	133.3 J/cm^2^
Application time per point	70 s
Number of points	12
Total energy	84 J
Application mode	Contact

**Figure 1:**
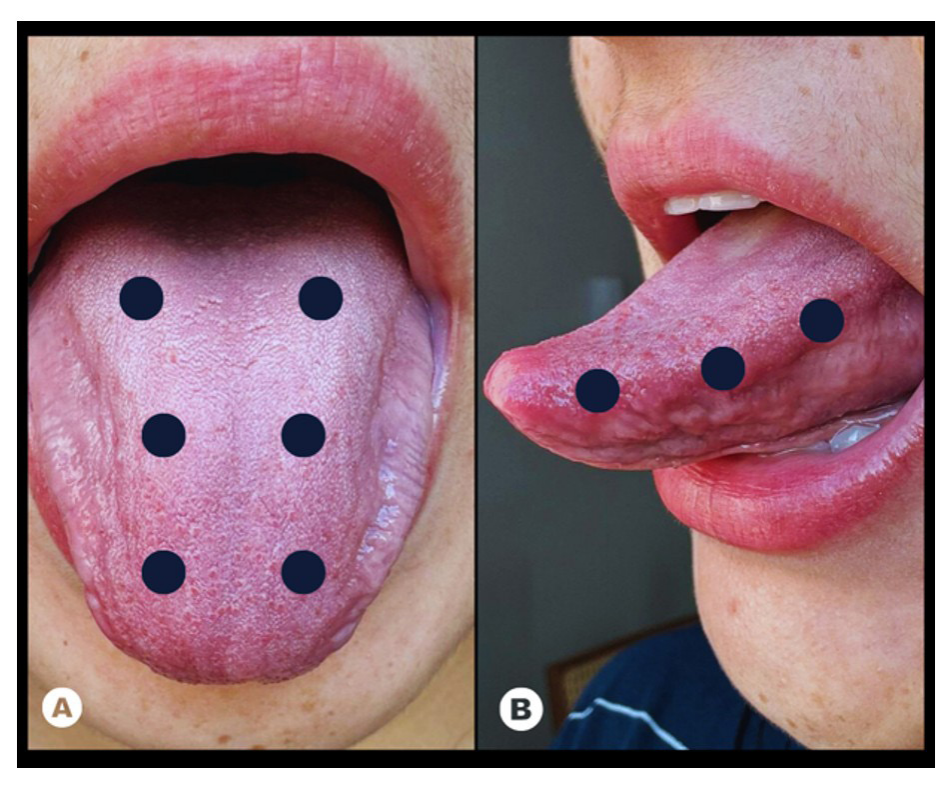
Laser application points on the tongue (A) upper and (B) lateral sides, respectively

The application was done with point contact at six points on the surface of the tongue and three points on the sides, bilaterally, as shown in Figure 1.

The application used 7, 9, and 11 J per point for 70, 90, and 110 seconds per point, respectively. PG underwent the same procedures as the irradiated groups, without activating the equipment.

The equipment tip was covered with plastic for sanitary purposes, being replaced after each participant. Researchers and participants wore protective eyewear provided by the manufacturers throughout the procedure.

The study was double-blind – i.e., neither the participants nor the researcher who conducted the assessments and reassessments knew which group each participant belonged to. Thus, the intervention they would receive was unknown, and they could not influence the outcome of the assessments.

After the laser application, the individuals rested for 10 minutes and were reassessed after this interval.

The data were analyzed for group homogeneity regarding age, maximum anterior and posterior tongue pressure, and anterior and posterior endurance before laser application using the Kruskal-Wallis test. Group homogeneity regarding sex was verified using the chi-square multiple comparison test. The Shapiro-Wilk test revealed that the data were not normally distributed. Therefore, the nonparametric Wilcoxon test was used to compare the outcomes (maximum anterior and posterior pressure and anterior and posterior endurance) before and after the intervention. It used the arithmetic mean of the three measurements of maximum anterior and posterior pressure for each parameter.

## RESULTS

The results indicated that the groups were homogeneous regarding sex (p = 0.896), age (p = 0.08), maximum anterior tongue pressure (p = 0.801), maximum posterior tongue pressure (p = 0.557), anterior endurance (p = 0.548), and posterior endurance (p = 0.396) before laser application.

Tables 1and 2 show the comparison of maximum anterior and posterior tongue pressure, respectively, before and after irradiation in each group, and of anterior and posterior tongue endurance, respectively, before and after irradiation in each group. There were no differences between before and after irradiation in any of the groups tested.

**Table 1:** Maximum anterior pressure (kPa) and anterior tongue endurance (s) before and after laser application

Group	G1 (n=15)				G2 (n=15)				G3 (n=15)				PG (n=15)			
	Anterior pressure		Anterior endurance		Anterior pressure		Anterior endurance		Anterior pressure		Anterior endurance		Anterior pressure		Anterior endurance	
	Before	After	Before	After	Before	After	Before	After	Before	After	Before	After	Before	After	Before	After
Mean	20.73	20.53	87.8	106.40	22.4	19.60	129.13	103.33	24.00	22.93	100.60	118.33	21.13	21.13	108.26	89.20
SD	7.48	10.97	76.65	96.98	4.37	8.54	81.30	70.53	13.70	15.36	83.59	85.27	7.00	6.83	89.43	71.32
Minimum	10.00	8.00	12.00	8.00	13.00	8.00	16.00	12.00	14.00	9.00	25.00	25.00	12.00	11.00	10.0	7.0
Maximum	33.00	46.00	315.00	271.00	28.00	42.00	269.00	234.00	68.00	75.00	249.00	298.00	36.00	37.00	303.00	235.00
p-value	0.454		0.290		0.077		0.418		0.504		0.493		0.933		0.648	

Wilcoxon test

Significance level of 5%

p-value ≤ 0.05

Caption: G1 = group irradiated with 7 J per point; G2 = group irradiated with 9 J per point; G3 = group irradiated with 11 J per point; PG = placebo group; SD = standard deviation

**Table 2:** Maximum posterior pressure (kPa) and posterior tongue endurance (s) before and after laser application

Group	G1 (n=15)				G2 (n=15)				G3 (n=15)				PG (n=15)			
	Posterior pressure		Posterior endurance		Posterior pressure		Posterior endurance		Posterior pressure		Posterior endurance		Posterior pressure		Posterior endurance	
	Before	After	Before	After	Before	After	Before	After	Before	After	Before	After	Before	After	Before	After
Mean	22.26	21.20	108.80	108.66	21.73	22.26	100.53	87.06	25.13	25.00	104.93	89.26	21.26	20.53	76.13	98.13
SD	10.36	11.85	98.05	78.72	7.37	12.51	52.64	54.80	10.39	13.51	103.54	79.63	8.45	9.23	79.24	89.93
Minimum	8.00	8.00	14.00	22.00	12.00	8.00	24.00	12.00	7.00	8.00	12.00	8.00	8.00	10.00	7.0	6.0
Maximum	42.00	51.00	356.00	317.00	39.00	60.00	227.00	213.00	52.00	65.00	426.00	265.00	42.00	38.00	315.00	360.00
p-value	0.677		0.724		0.818		0.442		0.574		0.633		0.560		0.395	

Wilcoxon test

Significance level of 5%

p-value ≤ 0.05

Caption: G1 = group irradiated with 7 J per point; G2 = group irradiated with 9 J per point; G3 = group irradiated with 11 J per point; PG = placebo group; SD = standard deviation

## DISCUSSION

The study aimed to compare the immediate effects of different doses of PBM with the 808 nm (infrared) wavelength on maximum tongue pressure and endurance. There are no studies in the literature addressing this muscle, and there is no consensus on the effects, dosimetry used, or even evidence regarding the ideal points for low-intensity laser application.

The results indicated no statistically significant difference between pre- and post-irradiation in any of the groups tested. It is believed that irradiation takes time to take effect. A study comparing the immediate effects of PBM with red and infrared wavelengths on the performance of the orbicularis oris muscle found no statistically significant differences in the parameters evaluated^(12)^.

Sex could have influenced the results, but statistical analysis revealed that the groups were homogeneously distributed; therefore, it was not a confounding factor. Baseline pressure and endurance were verified and compared to ensure that the groups were homogeneous, considering their initial strength and endurance capacity.

Research indicates that PBM therapy benefits muscle tissue, including improved muscle performance, increased strength, and muscle relaxation^(10,19)^. However, the findings of this study did not corroborate this idea, showing that, for the tongue, the 10-minute interval between irradiation and reassessments may have been insufficient to trigger muscle changes in strength and pressure. Another possible explanation for the lack of difference between before and after irradiation may have been its dose. One study found increased lip pressure after irradiation with 7 J^(10)^, which supported its choice for one of the study groups. However, the tongue's muscular organization is unique and distinct from that of the lips, which may have influenced the results. Furthermore, the genioglossus muscle plays an important role for the tongue to exert pressure on the palate, providing a stable platform and pressing the body of the tongue (intrinsic musculature) against the palate^(20)^. Because it is a deeper muscle, the genioglossus was certainly not affected by irradiation, which may have predominantly affected the intrinsic muscles.

Another possible explanation for the lack of difference between before and after irradiation is that the sample consisted of individuals without structural alterations observed through intraoral examination. It is possible that PBM balances muscle energy capacity only in individuals with alterations in these muscles. For future research, we suggest replicating the study for individuals with altered tongue muscles to investigate this hypothesis.

Lastly, the study findings demonstrated that PBM alone was unable to promote immediate changes in participants' maximum tongue pressure and endurance. However, the study had some limitations, such as the short interval between assessments before and after laser application and the absence of orofacial changes in the sample. This study innovates by evaluating the effects of laser on maximum tongue pressure and endurance, as no other study with these doses, application points, and outcomes was found. Much remains to be clarified about the PBM action mechanisms on muscle performance, and such studies are essential for understanding the effect of this resource. Thus, we suggest further research involving different doses and application points and pressure assessment in other tasks, combining orofacial myofunctional therapy, assessing differences between study groups, and including individuals with orofacial myofunctional alterations.

## CONCLUSION

This study found no statistically significant differences when comparing maximum tongue pressure and endurance before and after photobiomodulation.
