# Supplementary material for: Immediate effects of photobiomodulation on maximum pressure and endurance of the tongue in adults: a randomized clinical trial
Source: Codas. 2026 Jan 26;38(1):e20240317. doi: 10.1590/2317-1782/e20240317en (PMC12978336; doi:10.1590/2317-1782/e20240317en)
Supplement: Translation

# Translation: Efeitos imediatos da fotobiomodulação sobre a pressão máxima e resistência de língua em adultos: ensaio clínico randomizado

**DOI:** 10.1590/2317-1782/e20240317pt

**Authors:** Lara Kristine Silva Gomes Campos Soares, Lorena Freitas Coelho, Renata Maria Moreira Moraes Furlan, Mariana Rodrigues Batista

**Affiliations:** 1 Graduação em Fonoaudiologia, Universidade Vale do Rio Doce – UNIVALE - Governador Valadares (MG), Brasil.; 2 Departamento de Fonoaudiologia, Universidade Federal de Minas Gerais – UFMG - Belo Horizonte (MG), Brasil.; 3 Departamento de Fonoaudiologia, Universidade Vale do Rio Doce – UNIVALE - Governador Valadares (MG), Brasil.

**Keywords:** Força Muscular, Resistência Física, Terapia a Laser, Língua, Sistema Estomatognático

## Abstract

**Objetivo:**

Verificar se há efeitos imediatos da fotobiomodulação na pressão máxima e resistência da língua.

**Método:**

Trata-se de uma pesquisa experimental, duplo-cego, que investigou os efeitos imediatos da fotobiomodulação na pressão máxima e resistência da língua de sujeitos sem alterações funcionais desta estrutura. A amostra, não-probabilística, foi composta por 60 indivíduos, de ambos os sexos, com idade entre 18 e 35 anos, divididos em quatro grupos. As doses testadas foram 7, 9 e 11J por ponto, no comprimento de onda infravermelho, sendo aplicados em seis pontos na face superior da língua (sendo três pontos longitudinais, em duas fileiras) e três pontos longitudinalmente em cada lateral, totalizando 12 pontos de aplicação. O grupo placebo foi submetido aos mesmos procedimentos dos demais, porém, o equipamento não foi acionado. Os participantes foram submetidos a avaliação intraoral, parte da língua, do protocolo MBGR para incluir os indivíduos na amostra, além da avaliação da pressão máxima e resistência de língua utilizando o IOPI, pré e pós irradiação. Foram comparadas a pressão máxima e a resistência da língua antes e após a fotobiomodulação.

**Resultados:**

Os grupos foram homogêneos em relação ao sexo, idade, pressão máxima e resistência da língua antes da irradiação. Não houve diferença na pressão máxima ou na resistência da língua, entre os momentos pré e pós irradiação, em nenhum dos grupos testados.

**Conclusão:**

A fotobiomodulação, nas doses testadas, não provocaram efeitos imediatos na pressão ou na resistência da língua de adultos sem alterações estruturais e/ou funcionais da língua.

## INTRODUÇÃO

O sistema estomatognático é formado por estruturas que se relacionam de maneira complexa às funções de sucção, mastigação, deglutição, respiração e fala^(1)^. Estas são funções vitais do ser humano e qualquer alteração estrutural poderá gerar um desarranjo deste sistema como consequência^(2)^.

A língua é um órgão composto por músculos intrínsecos e extrínsecos que se ordenam de maneira a permitir, através de movimentos, a realização das funções orofaciais^(3,4)^.

Algumas condições clínicas comprometem a pressão e resistência muscular da língua, como por exemplo a Doença de Parkinson^(5)^, Respiração Oral^(6)^, Síndrome de Down^(7)^ e Esclerose Lateral Amiotrófica^(8)^. O fonoaudiólogo é o profissional que atua na prevenção, avaliação, diagnóstico e tratamento das alterações que possam acometer as funções estomatognáticas. De maneira crescente, o fonoaudiólogo tem se interessado em utilizar a fotobiomodulação (FBM) como recurso terapêutico^(9)^ por se tratar de uma técnica não invasiva, indolor, com baixo risco para o paciente, sem efeitos colaterais^(10)^ e sem relatos de toxicidade^(9)^. A FBM pode promover benefícios para o tecido muscular incluindo melhor desempenho muscular, incremento no ganho de força e relaxamento muscular^(11)^.

A FBM consiste na aplicação de luz a um sistema biológico capaz de estimular o início de um processo fotoquímico, visto de maneira mais ativa nas mitocôndrias, aumentando a produção de adenosina trifosfato (ATP)^(12)^, além de outros mecanismos moleculares de proliferação celular, aumentando interleucinas, síntese de ácido desoxirribonucleico (DNA), espécies de oxigênio reativas, citocromo c-oxidase, dentre outros^(13)^, favorecendo o metabolismo celular e podendo gerar efeitos tais como a analgesia, reparação tecidual, diminuição da fadiga muscular, dentre outros^(9)^.

Estudos recentes fizeram a investigação dos efeitos imediatos da FBM sobre a pressão exercida pelos lábios^(14)^ e sobre a fadiga eletromiográfica desta mesma musculatura^(10)^. Os resultados apresentaram incremento imediato da pressão exercida pelos lábios após serem submetidos a FBM no comprimento de onda de 808 nm, com a dosimetria de 7 J em seis pontos, ao redor dos lábios, com dose total de 42 J^(14)^. Não foram verificados efeitos sobre a fadiga eletromiográfica no músculo orbicular da boca, que utilizou a dose de 4 J por ponto com comprimentos de onda de 660 e 808 nm^(10)^.

A radiação é capaz de provocar efeitos fotoquímicos dentro de minutos e estes efeitos são conhecidos por efeitos imediatos^(15)^.

Não existem protocolos com dosimetria bem definida para cada caso, envolvendo a musculatura orofacial^(11)^, sendo o profissional que fará a terapia por fotobiomodulação que definirá qual comprimento de luz, qual a dose serão utilizadas, além de definir quais serão os pontos de irradiação. Diante disso, o profissional deverá entender os consequentes efeitos de cada dose, comprimento de onda e pontos de aplicação, tornando-se um desafio na utilização deste recurso terapêutico, não havendo consenso sobre os parâmetros ideais, bem como protocolos direcionados a cada objetivo^(11)^ na área de Motricidade Orofacial. Para que sejam criados protocolos direcionados envolvendo a fotobiomodulação e a musculatura orofacial, é necessário que mais estudos sejam realizados.

Existem poucos estudos sobre os efeitos da FBM na musculatura orofacial^(11,12)^, sendo que estes estudos focam, especialmente, o músculo orbicular da boca. Sabe-se que a fotobiomodulação tem efeitos fotoquímicos. Até o momento não existem estudos que investigaram os efeitos da fotobiomodulação sobre a musculatura da língua.

Diante disso, o objetivo principal da presente pesquisa foi verificar se há efeitos imediatos da fotobiomodulação na pressão máxima e resistência de língua.

## MÉTODOS

Trata-se de um ensaio clínico randomizado, duplo-cego, que investigou os efeitos imediatos da fotobiomodulação sobre a pressão máxima e resistência da língua em sujeitos sem alterações funcionais e/ou estruturais da língua.

A coleta de dados foi realizada na Universidade Vale do Rio Doce. O projeto foi aprovado pelo Comitê de Ética em Pesquisa da instituição (Parecer nº 6.854.040) e publicado no Registro Brasileiro de Ensaios Clínicos (ReBEC) com o número RBR-10bf7yj6.

A amostra, não probabilística, foi composta por 60 indivíduos sem alterações funcionais e/ou estruturais da língua, de ambos os sexos, com média de idade de 21,1 anos, mínimo de 18, máximo de 34 anos e desvio padrão de 2,9. Os participantes foram divididos, de maneira randomizada, por sorteio, em quatro grupos: grupo 1 (G1), grupo 2 (G2), grupo 3 (G3) e grupo placebo (GP). Os participantes pegavam um papel contendo um número dentro de uma caixa. Dentro desta caixa, estavam os números de 1 a 60, já definidos qual número pertencia a qual grupo de intervenção.

- Grupo 1 (G1): grupo submetido à fotobiomodulação com comprimento de onda de 808 nm (infravermelho), com dose de 7 J por ponto, totalizando 84 J.
- Grupo 2 (G2): grupo submetido à fotobiomodulação com comprimento de onda de 808 nm (infravermelho), com dose de 9 J por ponto, totalizando 108 J.
- Grupo 3 (G3): grupo submetido à fotobiomodulação com comprimento de onda de 808 nm (infravermelho), com dose de 11 J por ponto, totalizando 132 J.
- Grupo Placebo (GP): grupo submetido ao mesmo procedimento dos participantes do G1, G2 e G3, porém, o equipamento não foi acionado.

O tamanho amostral foi definido com base em estudos anteriores que avaliaram efeitos imediatos da fotobiomodulação em musculatura orofacial^(10-12)^, não tendo sido realizado o cálculo amostral. Foram convidados a participar do estudo alunos, professores e funcionários da instituição, que se encaixaram nos critérios de inclusão. Foram incluídos na amostra indivíduos de ambos os sexos, com idade entre 18 e 35 anos, que aceitaram participar da pesquisa e que assinaram o Termo de Consentimento Livre e Esclarecido (TCLE), com ausência de alterações cognitivas (era necessário que o participante conseguisse cumprir os comandos, realizando o que lhe era proposto. Caso o participante não tivesse capacidade de entendimento/cumprimento dos comandos, este não seria incluído), ausência de alterações funcionais e/ou estruturais da língua, ausência de lesões orais que acarretassem dor ou desconforto, frênulo lingual com fixações no terço médio da língua e nas carúnculas sublinguais (verificado por meio da parte de exame intraoral, especificamente da língua, do protocolo MBGR), ausência doenças neurogênicas (que tivessem impacto na compreensão ou cumprimento de comando por parte do participante) e que não apresentaram contraindicações para fototerapia. As contraindicações foram verificadas através de questionário, conforme manual dos fabricantes do equipamento e literatura específica, sendo elas fotossensibilidade, gravidez, glaucoma, lesão sem diagnóstico sobre a área a ser irradiada ou próxima a ela, infecção no local da aplicação, histórico de câncer, uso de marcapasso ou outro implante eletrônico. Também foram estabelecidos como critérios de inclusão: não utilizar medicamentos que levassem à fraqueza muscular^(16)^ no período de 48 horas que antecedeu a coleta dos dados e relatar alergia aos materiais usados. Esses dados foram coletados na entrevista inicial. Foram definidos como critérios de exclusão, a não execução de todas as tarefas propostas e a intolerância em manter o bulbo do *Iowa Oral Performance Instrument* (IOPI) na cavidade oral.

Na sequência, após a assinatura do TCLE, o participante foi orientado a permanecer sentado em uma cadeira, guiado pelo Plano de Frankfurt, mantendo flexão de 90º entre tornozelo, joelho e quadril, além da postura ereta. Foi realizada a parte de exame intraoral, especificamente da Língua do Protocolo de Avaliação Miofuncional – MBGR^(17)^, com o objetivo de identificar e excluir indivíduos com alterações desse órgão. A avaliação da língua foi feita por pesquisadora treinada e com experiência no tratamento dos distúrbios miofuncionais orofaciais.

A avaliação da pressão máxima e resistência foi feita por meio de um instrumento que apresenta valores numéricos para cada parâmetro avaliado. O instrumento utilizado foi o IOPI *,* que é constituído por um bulbo de ar (3,5 cm de comprimento e 1 cm de diâmetro), um transdutor de pressão, um tubo plástico de 1,5 cm que conecta o bulbo ao transdutor e uma tela de LCD. O bulbo do IOPI foi posicionado em duas regiões: primeiramente na região anterior da língua, logo atrás da papila alveolar e, em um segundo momento, em região posterior, com o seu limite anterior paralelo ao início dos primeiros molares^(18)^. Após posicionar o instrumento em região anterior, o participante foi solicitado a pressionar a língua em direção ao palato, com a maior força possível, pelo período de 2 segundos. Esse procedimento foi feito três vezes, com intervalo de 1 minuto e a pressão máxima foi o resultado da média aritmética de todos os picos máximos de pressão. Em seguida, o bulbo foi posicionado na região posterior da língua e o participante orientado a repetir o movimento, pelo mesmo tempo.

Para a avaliação da resistência da língua, o bulbo foi posicionado da mesma forma que na avaliação de pressão máxima da língua. Entretanto, o participante foi orientado a sustentar a força pelo tempo máximo que conseguisse. Para ter certeza da pressão que o participante estava exercendo, ele contou com o *biofeedback* do próprio IOPI, que acende uma luz verde quando o indivíduo atinge a pressão pré-programada (valor inserido pelo avaliador no IOPI que é a metade do valor de pressão máxima). Foi realizada uma coleta na região anterior e outra na região posterior da língua, com intervalo de 10 minutos entre as medições.

Após realizar a avaliação inicial, os participantes foram alocados de maneira aleatória (por meio de sorteio) nos grupos de intervenção. A aplicação da fotobiomodulação foi feita utilizando o equipamento da marca MMOptics®, modelo Laser Duo, de 100 mW de potência. Os parâmetros de irradiação encontram-se descritos no Quadro 1.

**Quadro 1:** Parâmetros do laser

**Parâmetros de irradiação**	**Valores**
Comprimento de onda	808 nm (infravermelho)
Modo de operação	Contínuo
Saída óptica	100 mW
Diâmetro de saída do feixe	1,95 mm
Área de saída do feixe	0,03 cm^2^
Densidade de potência	3,3 W/cm^2^
Energia por ponto	7J
Densidade de energia por ponto	133,3 J/cm^2^
Tempo de aplicação por ponto	70 s
Número de pontos	12
Total de energia	84 J
Modo de aplicação	Contato

A aplicação foi feita de modo pontual, com contato, em seis pontos sobre a superfície da língua e três pontos nas laterais, bilateralmente, conforme demonstrado na Figura 1.

**Figura 1:** Pontos de aplicação do laser sobre a língua (A) face superior e (B) face lateral, respectivamente

As doses utilizadas foram de 7, 9 e 11 J por ponto, sendo, respectivamente, o tempo de aplicação de 70, 90 e 110 segundos por ponto. O Grupo Placebo foi submetido aos mesmos procedimentos dos grupos irradiados, entretanto, o equipamento não foi acionado.

Para higienização foi utilizado plástico para encapar a ponteira do equipamento, sendo trocado a cada participante. Pesquisadores e participantes utilizaram óculos de proteção fornecidos pelos fabricantes durante todo o procedimento.

O estudo foi duplo-cego, sendo que a pesquisadora que realizou as avaliações e reavaliações dos participantes e os próprios participantes não sabiam sobre qual grupo aquele participante pertencia (sendo então desconhecida a intervenção que ele receberia, não podendo intervir no resultado das avaliações).

Após a aplicação do laser, os indivíduos foram submetidos a um descanso de 10 minutos, sendo reavaliados após este intervalo.

Os dados foram analisados quanto a homogeneidade dos grupos quanto à idade, pressão máxima anterior e posterior da língua, bem como quanto à resistência anterior e posterior, antes da aplicação do laser, utilizando o teste Kruskal Wallis. A homogeneidade dos grupos quanto ao sexo foi verificada pelo teste Qui-Quadrado de Comparações Múltiplas. Por meio do teste Shapiro-Wilk, verificou-se que os dados não apresentaram distribuição normal. Por isso, optou-se pela utilização do teste não paramétrico de Wilcoxon para as comparações dos desfechos (pressão máxima anterior e posterior e resistência anterior e posterior) entre os momentos pré e pós-intervenção. Para as pressões máxima anterior e posterior, utilizou-se a média aritmética das três medições realizadas para cada parâmetro.

## RESULTADOS

Os resultados indicaram que os grupos são homogêneos em relação ao sexo (p= 0,896), idade (p=0,08), pressão máxima anterior da língua (p= 0,801) e pressão máxima posterior da língua (p= 0,557), resistência anterior (p=0,548) e resistência posterior (p= 0,396) antes da aplicação do laser.

As Tabelas 1 e 2 apresentam a comparação da pressão máxima anterior e posterior da língua, respectivamente, antes e após a irradiação em cada grupo, além da comparação da resistência anterior e posterior da língua, respectivamente, antes e após a irradiação em cada grupo. Não houve diferença entre os momentos pré e pós irradiação em nenhum dos grupos testados.

**Tabela 1:** Pressão anterior máxima (kPa) e resistência anterior da língua (s) antes e após a aplicação do laser

Grupo	G7 (n=15)				G9 (n=15)				G11 (n=15)				GP (n=15)			
	Pressão anterior		Resistência anterior		Pressão anterior		Resistência anterior		Pressão anterior		Resistência anterior		Pressão anterior		Resistência anterior	
	Pré	Pós	Pré	Pós	Pré	Pós	Pré	Pós	Pré	Pós	Pré	Pós	Pré	Pós	Pré	Pós
Média	20,73	20,53	87,8	106,40	22,4	19,60	129,13	103,33	24,00	22,93	100,60	118,33	21,13	21,13	108,26	89,20
DP	7,48	10,97	76,65	96,98	4,37	8,54	81,30	70,53	13,70	15,36	83,59	85,27	7,00	6,83	89,43	71,32
Mínimo	10,00	8,00	12,00	8,00	13,00	8,00	16,00	12,00	14,00	9,00	25,00	25,00	12,00	11,00	10,0	7,0
Máximo	33,00	46,00	315,00	271,00	28,00	42,00	269,00	234,00	68,00	75,00	249,00	298,00	36,00	37,00	303,00	235,00
Valor de p	0,454		0,290		0,077		0,418		0,504		0,493		0,933		0,648	

Teste de Wilcoxon

Nível de significância de 5%

valor de p≤0,05

Legenda: G7 = grupo irradiado com 7 J por ponto; G9 = grupo irradiado com 9 J por ponto; G11 = grupo irradiado com 11J por ponto; GP = grupo placebo; DP = desvio padrão

**Tabela 2:** Pressão posterior máxima (kPa) e resistência posterior da língua (s) antes e após a aplicação do laser

Grupo	G7 (n=15)				G9 (n=15)				G11 (n=15)				GP (n=15)			
	Pressão posterior		Resistência posterior		Pressão posterior		Resistência posterior		Pressão posterior		Resistência posterior		Pressão posterior		Resistência posterior	
	Pré	Pós	Pré	Pós	Pré	Pós	Pré	Pós	Pré	Pós	Pré	Pós	Pré	Pós	Pré	Pós
Média	22,26	21,20	108,80	108,66	21,73	22,26	100,53	87,06	25,13	25,00	104,93	89,26	21,26	20,53	76,13	98,13
DP	10,36	11,85	98,05	78,72	7,37	12,51	52,64	54,80	10,39	13,51	103,54	79,63	8,45	9,23	79,24	89,93
Mínimo	8,00	8,00	14,00	22,00	12,00	8,00	24,00	12,00	7,00	8,00	12,00	8,00	8,00	10,00	7,0	6,0
Máximo	42,00	51,00	356,00	317,00	39,00	60,00	227,00	213,00	52,00	65,00	426,00	265,00	42,00	38,00	315,00	360,00
Valor de p	0,677		0,724		0,818		0,442		0,574		0,633		0,560		0,395	

Teste de Wilcoxon

Nível de significância de 5%

valor de p≤0,05

Legenda: G7 = grupo irradiado com 7 J por ponto; G9 = grupo irradiado com 9 J por ponto; G11 = grupo irradiado com 11J por ponto; GP = grupo placebo; DP = desvio padrão

## DISCUSSÃO

O estudo teve como objetivo comparar os efeitos imediatos de diferentes doses da fotobiomodulação com o comprimento de onda de 808 nm (infravermelho) sobre a pressão máxima e resistência de língua, tendo em vista que na literatura não há estudos que abordaram essa musculatura, não havendo consenso sobre os efeitos, a dosimetria utilizada e nem mesmo evidências sobre os pontos ideais da aplicação do laser de baixa intensidade.

Os resultados indicaram que não houve diferença estatisticamente significativa entre os momentos pré e pós irradiação em nenhum dos grupos testados. Acredita-se que a irradiação leva um tempo para fazer efeito. Um estudo que comparou os efeitos imediatos da fotobiomodulação nos comprimentos de onda vermelho e infravermelho no desempenho do músculo orbicular da boca, não encontrou diferenças estatisticamente significativas sobre os parâmetros avaliados^(12)^.

A variável sexo poderia interferir nos resultados, mas após análise estatística foi constatado que os grupos estavam com distribuição homogênea, não sendo, então, um fator de confusão. Foram verificadas e comparadas as pressões e resistências de base para garantir que os grupos fossem homogêneos, considerando a capacidade inicial de realizar a força e resistência.

As pesquisas apontam que são benefícios da terapia por fotobiomodulação para o tecido muscular melhor desempenho muscular, maior ganho de força e relaxamento da musculatura^(10,19)^. No entanto, os achados da presente pesquisa não corroboraram esta ideia, mostrando que, para a língua, o tempo de 10 minutos entre a irradiação e as reavaliações podem ter sido insuficientes para que as mudanças musculares, no que se refere a força e pressão, começassem a ocorrer. Outra possível explicação para a ausência de diferença entre os momentos pré e pós irradiação pode ter sido a dose utilizada. Uma pesquisa verificou aumento da pressão de lábios após irradiação com 7 J^(10)^, o que subsidiou a escolha desta dose para um dos grupos deste estudo, mas a organização muscular da língua é única e distinta da dos lábios, o que pode ter influenciado os resultados. Além disso, para que a língua exerça pressão no palato, o musculo genioglosso tem importante atuação, fornecendo uma plataforma estável e pressionando o corpo da língua (musculatura intrínseca) conta o palato^(20)^. Por ser um músculo mais profundo, certamente o genioglosso não foi atingido pela irradiação, que pode ter atingido predominantemente os músculos intrínsecos.

Mais uma possível explicação para a ausência de diferença entre os momentos pré e pós irradiação reside no fato de que a amostra foi composta por indivíduos sem alterações estruturais observadas a partir do exame intraoral. É possível que a fotobiomodulação atue de forma a equilibrar a capacidade energética muscular apenas para indivíduos com alterações nessa musculatura. Sugere-se, para futuras pesquisas, que o estudo seja replicado para indivíduos com alteração da musculatura da língua, a fim de investigar essa hipótese.

Por fim, os achados dessa pesquisa demonstraram que, isoladamente e de maneira imediata, a fotobiomodulação não foi capaz de promover modificações com relação à pressão máxima e resistência da língua dos participantes. No entanto, o estudo apresentou algumas limitações, como, por exemplo, o intervalo curto entre as avaliações pré e pós aplicação do laser e a ausência de alterações orofaciais na amostra. A presente pesquisa inova ao avaliar os efeitos do laser na pressão máxima e na resistência da língua, não tendo sido encontrado outro estudo com estas doses, pontos de aplicação e estes desfechos. Ressalta-se que há muito o que ser esclarecido sobre os mecanismos de ação da fotobiomodulação no desempenho muscular e que estes estudos são primordiais para se compreender o efeito deste recurso. Para isso, sugere-se que demais pesquisas sejam realizadas, envolvendo diferentes doses, diferentes pontos de aplicação, avaliação de pressão em outras tarefas, associando a terapia miofuncional orofacial e avaliando se há diferença entre grupos pesquisados, além de abranger indivíduos com alterações miofuncionais orofaciais.

## CONCLUSÃO

O presente estudo não encontrou diferenças estatisticamente significativas ao comparar a pressão máxima e resistência de língua pré e pós fotobiomodulação.
